# The inventory of psychotic-like anomalous self-experiences (IPASE): Stability and relationships with attenuated psychotic symptoms and remission in individuals at-risk for psychosis

**DOI:** 10.1016/j.schres.2025.05.003

**Published:** 2025-05-28

**Authors:** Isabelle Scott, Alexandra Selloni, Zarina Bilgrami, Matthew Cotter, Cansu Sarac, Alessia McGowan, Marija Krcmar, Melanie Formica, Kate Gwyther, Cassandra Wannan, Agrima Srivastava, Guillermo A. Cecchi, Romina Mizrahi, Patrick McGorry, Cheryl M. Corcoran, Barnaby Nelson

**Affiliations:** a Centre for Youth Mental Health, The University of Melbourne, Parkville, Melbourne, Victoria, Australia; b Orygen, The National Centre for Excellence in Youth Mental Health, 35 Poplar Rd, Parkville, VIC, Australia; c Icahn School of Medicine at Mount Sinai, New York, NY, USA; d Emory University, Atlanta, Georgia; e IBM TJ Watson Research Center, Yorktown Heights, NY, USA; f Douglas Research Centre, McGill University, Montreal, Quebec, Canada; g Department of Psychiatry, McGill University, Montreal, Quebec, Canada

## Abstract

**Background:**

The IPASE is a self-report measure of basic self-disturbance, a core feature of schizophrenia and ultra-high risk (UHR) states. However, the extent to which basic self-disturbance—as captured by the IPASE—is stable over time and related to the severity or progression of attenuated psychotic symptoms (APS) remains unclear. We examined the temporal stability of IPASE scores, their correlation with APS, and whether they predict changes in APS over time.

**Methods:**

The baseline sample included 185 participants (healthy controls = 72, UHR = 66, first-episode psychosis = 47), with 29 UHR participants re-assessed at month-12. Correlations between IPASE scores and Comprehensive Assessment of At-Risk Mental States (CAARMS) positive symptom scores were evaluated at baseline and month-12. Stability between baseline and month-12 IPASE scores was examined in the longitudinal subsample. Regression was used to predict remission and change in CAARMS scores.

**Results:**

Although mean IPASE scores were significantly higher in the UHR group compared to HCs, total IPASE scores were only weakly correlated with CAARMS total scores (*ρ*=0.27). IPASE subscales showed weak correlations (0.08<*ρ*<0.27) with CAARMS positive symptom domains. Changes in IPASE and CAARMS scores were not correlated. Moderate stability was found for IPASE total scores (ICC = 0.59) and four subscales (0.58 < ICC < 0.64), excluding the cognition subscale (ICC = 0.3). Baseline IPASE scores did not predict remission (partial *R*^2^ =0.05) or change in CAARMS scores (partial *R*^2^ =0.02).

**Conclusion:**

The IPASE is a moderately stable measure in UHR individuals, correlates with the presence of positive psychotic symptoms but only weakly with severity, and does not strongly predict positive symptom change.

## 1. Introduction

Basic self-disturbance is a core feature of schizophrenia spectrum disorders and has also been identified in individuals at ultra-high risk (UHR) for psychosis (Henriksen et al., 2021; Nordgaard et al., 2021; Parnas, 2011; Parnas et al., 2011; Sass, 2001; Nelson et al., 2012). The ‘basic self’ refers to the pre-reflective and immediate consciousness of action, experience and thought (see Feyaerts et al. (2024) for critical discussion of the concept). It captures the ‘first person’ sense of ownership of experience and agency of action (Gallagher, 2011), which are generally implicit aspects of normal basic selfhood and facilitate interactions with others/the world (Kircher and David, 2003). Instability of the basic self can manifest in a variety of anomalous subjective experiences, which can intensify and crystallise over time into positive and negative psychotic symptoms (Fuchs, 2015).

Phenomenological interviews and self-report scales have been used to measure basic self-disturbance. The Examination of Anomalous Self Experience (EASE) (Parnas et al., 2005), a semi structured qualitative interview, is regarded as the ‘gold standard’ measure of anomalous self-experiences. It has shown high internal consistency and interrater reliability, and relative specificity to the schizophrenia spectrum (Nordgaard et al., 2021). Additionally, the EASE interview has demonstrated that anomalous self-experiences exist during the early stages of schizophrenia as well as amongst individuals who are at ultra-high risk (UHR) for psychosis, in whom they predict later onset of psychosis (Nelson et al., 2012; Nordgaard et al., 2021). Furthermore, both baseline and change in total EASE scores to follow-up were found to be a predictor of remission in individuals with schizophrenia and other psychotic disorders at 7-year follow-up (Svendsen et al., 2019).

Basic self-disturbance has generally been conceptualised as an enduring, “trait-like” feature, rather than dynamic, “state-like” characteristic (Parnas, 2012; Parnas et al., 2005). However, there has also been theoretical discussion that some phenomena associated with self-disturbance may be more reactive or state-like, constituted by interactions between environmental factors and individual vulnerabilities (Borda & Sass, 2015; Sass et al., 2018). The notion that basic self-disturbance is trait-like is supported by empirical findings that anomalous self-experiences are moderately stable (Nordgaard et al., 2021). At 7-year follow-up in non-psychotic help-seeking adolescents, test-retest total EASE scores were found to be moderately correlated (*ρ*=0.64). However, there was variability amongst the subscales (0.04<*ρ*<0.64), with a negligible test-retest correlation for both the demarcation/transitivism and existential reorientation domains (Koren et al., 2020). Similarly, total scores from a 25-item self-disorder scale, considered as a precursor to the EASE, were also found to be moderately correlated (*ρ* = 0.64) at 5-year follow up in participants with schizophrenia-spectrum disorders (Nordgaard et al., 2017).

While the EASE is considered to be the gold standard for the assessment of anomalous self-experiences, it requires comprehensive training for both administration and scoring. Therefore, a self-report scale, the Inventory of Psychotic-Like Anomalous Self-Experiences (IPASE), was developed (Cicero et al., 2017). While the IPASE was informed by the EASE, its items do not represent a direct translation of items within the EASE (Cicero et al., 2017), and as such, the IPASE should not be viewed as a shortened self-report version of the EASE. The IPASE has been shown to have a high internal reliability (Cronbach’s α of 0.96–0.98) (Cicero et al., 2017) and construct validity for basic self-disturbance (Nelson et al., 2019). IPASE total scores were found to strongly correlate with EASE total (Pearson’s *r* = 0.92) and CAARMS positive symptom (Pearson’s *r* = 0.74) scores in a previous study involving healthy control, UHR and first-episode psychosis (FEP) participants (Nelson et al., 2019).

Despite correlations between the EASE and IPASE, and the shared purpose of these instruments in measuring ASEs, key differences between these instruments indicate that psychometric properties should not be assumed to transfer. As a self-report questionnaire, the IPASE may differ in important ways from the clinician-administered EASE — for example, in its susceptibility to participant interpretation, insight, or distress (Michel et al., 2017). Additionally, the IPASE demonstrates a five-factor structure (Cicero et al., 2017), in contrast to the unifactorial structure typically observed for the EASE (Nordgaard & Parnas, 2014; Nordgaard et al., 2023), supporting the need for independent psychometric evaluation. Such differences are unsurprising as the IPASE was not designed as a direct replication of the EASE, but a scalable alternative informed by the EASE (Cicero et al., 2017). As such, it is important to independently examine whether ASEs, as measured by the IPASE, demonstrate the same level of temporal stability as has been reported for the EASE. Further, whether IPASE scores are a predictor of the progression or remission of attenuated psychotic symptoms, which, to our knowledge, has not been investigated to date.

The aims of the present study were to: (1) Confirm previous findings of an association between anomalous self-experiences, as measured by IPASE total scores, and total positive symptoms as measured by the CAARMS. We also extended these findings to examine specific associations between IPASE subscales and CAARMS positive symptom categories. (2) Investigate the stability of IPASE measurements over time. (3) Determine whether baseline IPASE measures predicted progression of attenuated psychotic symptoms in the UHR group, both categorically (remission or persistence of APS) and continuously (changes in CAARMS total positive symptom scores). To achieve these aims, we examined the agreement and correlation of IPASE scores at two separate time points for a subgroup of UHR individuals who were followed prospectively. In contrast to previous studies that examined the stability of anomalous self-experiences with 5 (Nordgaard et al., 2017) and 7 (Koren et al., 2020) year follow-ups, we examine the stability of IPASE scores between baseline and 12 months. To predict UHR remission or change in CAARMS scores, multiple regression analysis was conducted with baseline CAARMS and IPASE scores as predictors. Based on previous test-retest analyses of the EASE, we hypothesised that the IPASE would exhibit moderate stability over time but with variability across domains. We further hypothesised that the demarcation/transitivism domain of the IPASE would show low stability, consistent with stability findings for the EASE. Finally, based on prior studies using the EASE (Comparelli et al., 2016; Nelson et al., 2012; Raballo et al., 2016; Værnes et al., 2021), we hypothesised that IPASE scores would be moderately correlated with CAARMS positive symptoms, and that lower IPASE scores would be a significant predictor of remission and larger reductions in CAARMS total positive symptoms from baseline to month-12.

## 2. Material and methods

### 2.1. Participants

The sample consisted of a subset of participants enrolled in the SPEAK study who were recruited at Orygen, a youth mental health service in northwestern Melbourne, Australia. As part of the inclusion criteria (for assessment purposes), all participants spoke adequate English and had an IQ of 80 or greater, as measured by the Wechsler Abbreviated Scale of Intelligence (WASI; Wechsler, 1999). Baseline analysis included all 185 individuals from this site who completed a baseline assessment, who were a mixture of healthy control, UHR, and first-episode psychosis individuals (HC = 72, UHR = 66, FEP = 47). All other analyses included a further subset of 29 UHR individuals for whom longitudinal clinical assessments were also conducted. The remaining 37 UHR individuals were lost to follow-up as a result of challenges related to the Covid-19 pandemic. Additionally, the primary aim of the SPEAK study was baseline characterisation of the sample, and as such, participants were included even if they were unable to complete a follow-up assessment. A full description of the study protocol and participants is described elsewhere (Bayer et al., 2023). Participants were assessed using the CAARMS, and completed the IPASE self-report questionnaire, all at baseline and for a UHR subset at 12 months. All 29 individuals were evaluated as clinical high risk for psychosis at baseline using standard diagnostic criteria based on the CAARMS (Nelson, 2014; Yung et al., 2005). The 29 participants who were followed up received psychosocial care that included case management, symptom monitoring, and individual psychotherapy (type and duration varied). Some also received antidepressant medication. No individuals received antipsychotic medication during the study period.

### 2.2. Measures

The Inventory of Psychotic-Like Anomalous Self-Experiences (IPASE) is a 57-item self-report questionnaire. Participants rate on a scale of 1 (Strongly Disagree) to 5 (Strongly Agree) how much they agree with statements relating to anomalous self-experiences. The IPASE contains 5 subscales: Self-Awareness and Presence, Consciousness, Somatization, Cognition and Demarcation/Transitivism. Subscale scores are calculated as the sum of the ratings for each item in the subscale (see supplementary material Table S1 for a list of items corresponding to each subscale). All participants were given the opportunity to ask clarifying questions to the research assistant if they were unsure of the interpretation of any items.

The Comprehensive Assessment of At Risk Mental States (CAARMS) (Yung et al., 2005) is a semi-structured interview used to identify help-seeking young people who are at UHR of developing psychosis and to track and quantify the severity of psychopathology over time. The CAARMS was also administered to HC and FEP participants. The CAARMS consists of seven domains: positive symptoms, cognitive change, emotional disturbance, negative symptoms, behavioural change, motor/physical changes, and general psychopathology. The positive symptoms domain includes items related to four categories: (1) unusual thought content, (2) non-bizarre ideas, (3) perceptual abnormalities, and (4) disorganised speech. The intensity and frequency of positive symptoms are rated on a scale of 0–6 by the assessor, and are used to identify individuals at UHR for psychosis and those exhibiting full-threshold psychosis. Individuals may also be identified as UHR if they meet vulnerability criteria (e.g. family history) and have had a decline in functioning, as measured by the SOFAS (Social and Occupational Functioning Assessment) (Morosini et al., 2000; Yung et al., 2005). Total positive symptom scores are calculated as the sum of intensity by frequency scores across each positive symptom item (1.1–1.4) (Morrison et al., 2012). It was decided to use the CAARMS instead of the Positive and Negative Symptom Scale (PANSS) (Kay et al., 1987) because the FEP participants in this study are treated FEP patients who primarily have residual symptoms and low PANSS scores. Therefore, if the PANSS was used, there would not be enough variance in the PANSS scores for FEP participants or for UHR participants, who have attenuated symptoms. The CAARMS was used to better capture variance in symptoms.

### 2.3. Statistical analysis

All analyses were performed using R version 4.3.0 (Core Team and R., 2021).

#### 2.3.1. Correlation between CAARMS and IPASE scores

Spearman’s *ρ* was used to assess the correlation between total IPASE and total positive CAARMS scores, and between the five IPASE and four CAARMS positive symptom subscales, at baseline for the larger sample of 185 individuals. The correlation between total IPASE and total positive CAARMS scores was also assessed at baseline and month-12 for the smaller subsample of 29 UHR individuals with longitudinal measurements.

#### 2.3.2. Longitudinal analyses

UHR participants were divided into those who remitted symptomatically and those who exhibited persistent attenuated positive symptoms:

1. Symptomatic remission: participants who did not meet UHR criteria, according to the CAARMS, at 12-month follow-up.
2. Persistent attenuated positive symptoms: participants who met UHR criteria, according to the CAARMS, at 12-month follow-up.

Given the small sample size of the longitudinal subsample, non-parametric tests were used to assess group differences. Wilcoxon rank-sum (Mann-Whitney) tests (Mann and Whitney, 1947; Wilcoxon, 1992) were used to compare the mean CAARMS total positive symptom score and mean total IPASE score between the remission and persistent APS groups at baseline and month-12. Wilcoxon signed-rank tests (Wilcoxon, 1992) were used to compare the mean CAARMS total positive symptom score and mean total IPASE score between baseline and month-12 for both the remission and persistent APS group.

The intraclass correlation coefficient (ICC) was used to measure the agreement between IPASE scores at baseline and month-12. The ICC was calculated for both IPASE total and subscale scores. The ICC agreement is an appropriate metric to measure the concordance between repeated measures in this setting, given that the IPASE is proposed to measure a single trait-like construct, self-disturbance (Liu et al., 2016). However, Spearman’s rho was also calculated to facilitate comparison with previous stability analyses of the EASE (Koren et al., 2020; Nordgaard et al., 2017). Bootstrapped 95 % confidence intervals were calculated for Spearman’s rho using the RVAideMemoire package version 0.9–83-22.

Regression analyses for the longitudinal sample were performed using the R stats package version 4.3.0. Multiple logistic regression analyses were performed to predict remission whilst multiple linear regression was used to predict the change in CAARMS total positive symptoms scores from baseline to month-12. For both models, the dependent variables were regressed on CAARMS total positive symptom and IPASE total scores at baseline. To address the risk of Type 2 error due to the small sample size, we also tested for moderate effect sizes using partial *R*^2^(small = 0.01, medium = 0.06, large = 0.14) (Fritz et al., 2012), which measures the proportion of variance in the dependent variable attributable to each predictor.

## 3. Results

### 3.1. Demographic and clinical characteristics

The demographic and clinical characteristics of the full baseline sample and longitudinal subsample, measured at baseline, are provided in Table 1. The UHR and FEP groups were, on average, 1–2 years younger and had a lower level of education than the healthy control group. The FEP group had the highest positive and negative symptoms, IPASE scores, and poorest functioning. The demographic and clinical characteristics of the longitudinal subsample of UHR individuals were similar to those calculated for the entire baseline sample of UHR individuals. No individuals from the longitudinal subsample were taking antipsychotic medication. There were no significant differences in total IPASE scores (*p* = 1.00) or total CAARMS positive symptom scores (*p* = 0.89) between UHR individuals included in the follow-up analyses and those lost to follow-up.

**Fig. 1.**
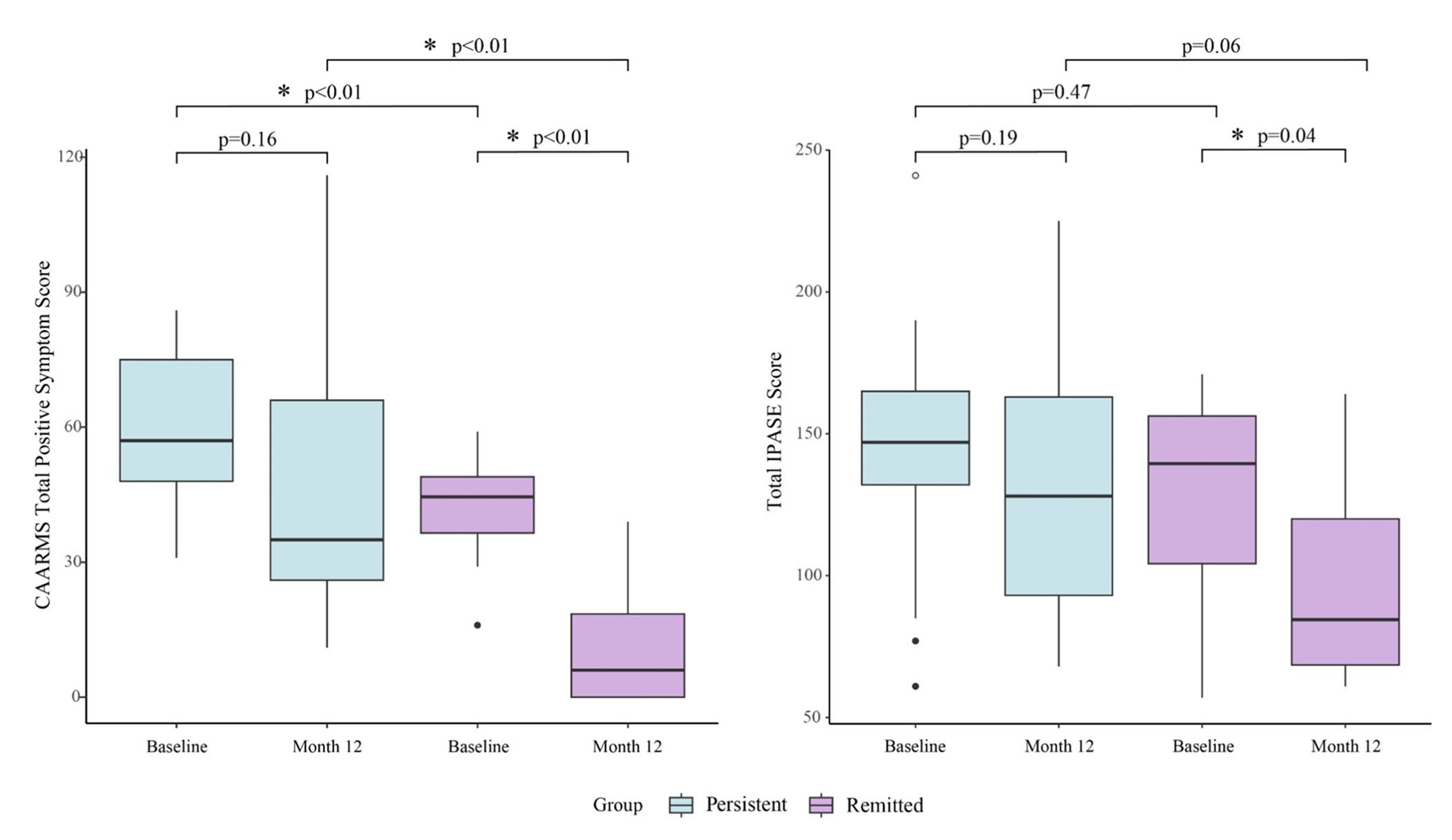
CAARMS total positive symptom scores (left) and total IPASE scores (right) for longitudinal subsample, divided into persistent APS (pink) and remitted (blue) subgroups. Outliers displayed as hollow bullet points. Wilcoxon rank-sum and Wilcoxon signed-rank tests were used to determine group differences. * Denotes a significant (*p* < 0.05) difference between groups. (For interpretation of the references to colour in this figure legend, the reader is referred to the web version of this article.)

The mean CAARMS total positive symptom scores and total IPASE scores for the longitudinal sample are displayed in Fig. 1, with the baseline scores for the longitudinal sample also displayed in Table 1. There was a significant difference in the mean CAARMS total positive symptom scores between the persistent APS (*n* = 17) and remission (*n* = 12) groups at baseline and month-12. As expected, there was also a significant reduction in CAARMS total positive symptom scores between baseline and month-12 for the remission group but not for the persistent APS group. The persistent APS group exhibited a smaller non-significant reduction in CAARMS total positive symptom scores. There was no significant difference in the mean total IPASE scores between the persistent APS and remission groups at baseline. The mean total IPASE scores decreased significantly only for the remission group between baseline and month-12. However, there was an observable non-significant difference in means between the two time points for the persistent APS group, and between the persistent and remitted group at month-12. There were no differences in IPASE findings (at *P* = 0.05 level of significance) after removal of the outlier present in the persistent APS group.

### 3.2. Relationship between CAARMS and IPASE scores

**Fig. 2.**
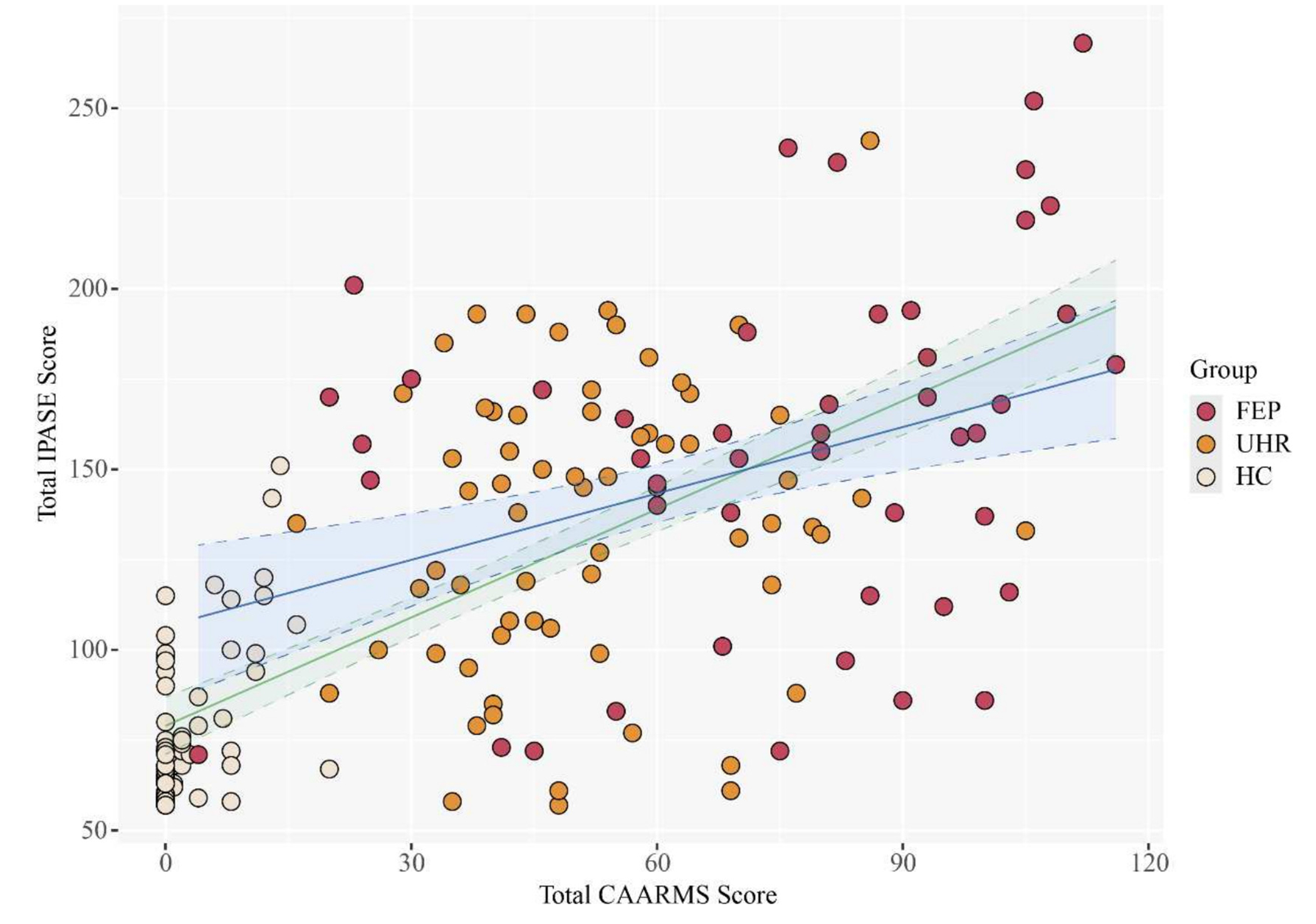
Scatterplot of the relationship between the IPASE total and CAARMS total positive symptoms scores for baseline sample. Correlation between total scores excluding (blue) and including (green) healthy controls. (For interpretation of the references to colour in this figure legend, the reader is referred to the web version of this article.)

There was a weak positive correlation (*ρ*=0.27, 95 % CI 0.08–0.44) between the total IPASE and CAARMS total positive symptom scores for the baseline sample of UHR and FEP participants (Fig. 2). However, with inclusion of the healthy control subgroup, this increased to a strong positive correlation (*ρ*=0.71, 95 % CI 0.63–0.78). When analysed separately, the correlation between total IPASE and CAARMS total positive symptom was slightly higher for the FEP (*ρ*=0.36, 95 % CI 0.03–0.61) than the UHR subgroup (*ρ*=0.18, 95 % CI −0.04–0.40). Across the baseline sample of UHR and FEP participants, each of the five IPASE subscales exhibited only a weak or very weak correlation (0.08<*ρ*<0.27) with the four CAARMS positive symptom subscales. No correlations remained statistically significant (*p* < 0.05) after applying the Holm adjustment. Conversely, when the healthy control group was again included, a significant moderate-strong correlation (0.48<*ρ*<0.61) was found between all subscales (supplementary material Table S2).

**Fig. 3.**
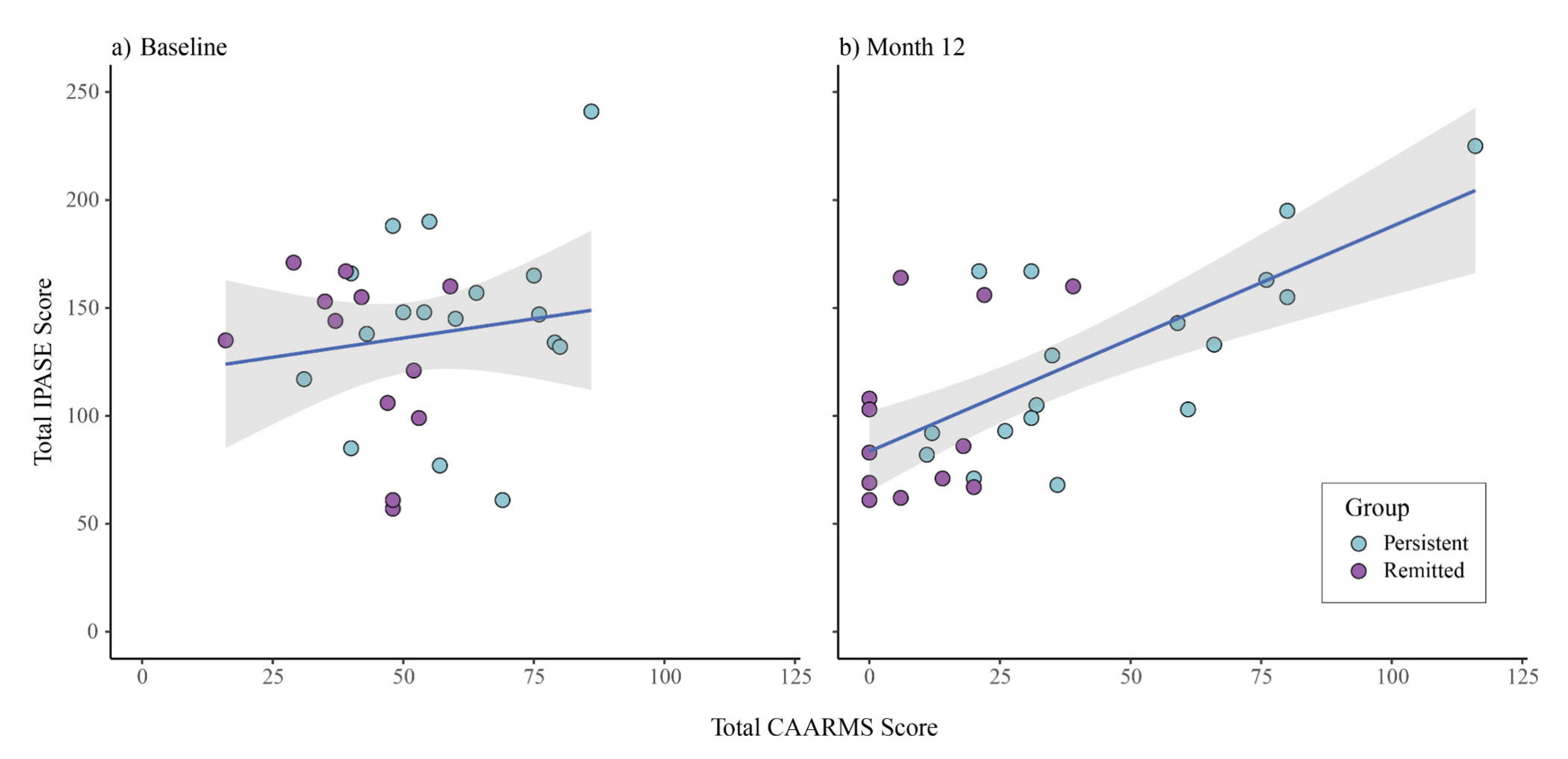
Scatterplot of the relationship between the IPASE total and CAARMS total positive symptoms scores at a) baseline and b) month-12 follow-up. Persistent individuals (blue) met UHR criteria at month-12 follow-up. Remitted individuals (pink) did not meet UHR criteria at follow-up. (For interpretation of the references to colour in this figure legend, the reader is referred to the web version of this article.)

When analysing only the longitudinal subsample, there was no correlation between the total IPASE and total positive symptom CAARMS scores at baseline (*ρ*=0.02, 95 % CI −0.35–0.40), but there was a strong positive correlation at 12 months (*ρ*=0.58, 95 % CI 0.28–0.80) (Fig. 3). Sensitivity analysis conducted by removing the outlier (Fig. 1) did not indicate any qualitative differences in this relationship at baseline (*ρ*=0.02) or month-12 (*ρ*=0.54). At baseline (Fig. 3a), all participants met UHR criteria, whilst at month-12, 12 of the 29 individuals remitted, leading to a larger variance in the CAARMS values at this time point (Fig. 3b). For the longitudinal subsample, despite a significant reduction in both CAARMS total positive symptom scores and total IPASE scores for the remission group, the change in these two scores between baseline and month-12 was not significantly correlated (*ρ*=0.08, 95 % CI −0.36–0.46).

### 3.3. Stability of IPASE scores

The test-retest absolute agreement between baseline and month-12 total IPASE scores was moderately high (ICC = 0.59, *p* < 0.001)(Table 2). The correlations between baseline and month-12 scores was moderate across four of the five subscales (0.54 < ICC < 0.64), but poor for the Cognition subscale. However, the confidence intervals for all ICC values were wide. For the IPASE total scores, and all subscales excluding cognition, the confidence intervals encompassed values corresponding to a weak or strong correlation (CIs within the range 0.20–0.88). In the case of cognition, the confidence interval included negative and moderate ICC values (−0.11–0.61). The Spearman correlations were consistent with the ICC results. Across all IPASE subscales, the Demarcation/Transitivism subscale exhibited the highest ICC and Spearman’s correlation.

### 3.4. IPASE scores as a predictor of attenuated psychotic symptoms

Multiple logistic regression analysis indicated that baseline CAARMS total positive symptom scores had a small but significant negative impact on the odds of remission (−10 % reduction in odds of remission, *p* = 0.02) and explained a moderate proportion of the variance in outcome (Partial *R*^2^= 0.30) (Table 3). Conversely, baseline IPASE total scores were not found to be a significant predictor of remission (2 % increase in odds of remission, *p* = 0.12) and only explained a small proportion of the variance in outcome (Partial *R*^2^= 0.05). The full model was preferred over an intercept only model (LRT = 12.06, *p* < 0.01). However, likely due to the small sample size, diagnostic assessments (supplementary material Fig. S1) indicated only an average fit of the model to the data with >5 % of residuals falling outside the expected 95 % confidence intervals (Gelman et al., 2000).

Multiple linear regression analysis indicated that neither the baseline CAARMS total positive symptom scores nor the baseline IPASE total scores were significant predictors of the change in CAARMS scores between baseline and month-12. Both predictors only explained a small proportion of the variance in outcome. In this case, the intercept only model was preferred over the full model (LRT = 1454.58, *p* = 0.35). Diagnostic assessments indicated an acceptable model fit after removal of one influential outlier (supplementary material Fig. S2). Sensitivity analysis demonstrated no qualitative change to the significance of predictors or effect size (supplementary material Table S3) after removal of the outlier. The intercept only model was still preferred over the full model (LRT = 1600.10, *p* = 0.27).

The variance inflation factor for both regression models was less than two, indicating that the models were not affected by multicollinearity between predictors (baseline IPASE and CAARMS total positive symptoms scores).

## 4. Discussion

In contrast to our hypothesis, there was only a weak positive correlation between IPASE total and CAARMS total positive symptom scores across the baseline clinical sample (UHR = 66, FEP = 47). However, a strong positive correlation was observed when healthy controls were included (HC = 72, UHR = 66, FEP = 47). This finding is consistent with previous work in which a strong positive correlation between IPASE total and CAARMS total positive symptom scores was found amongst a smaller sample of 49 individuals that also included healthy controls (HC = 11, UHR = 21, FEP = 14) (Nelson et al., 2019). In the present study, only weak non-significant pairwise correlations were found between each of the four CAARMS positive symptoms subscales and the five IPASE subscales (UHR = 66, FEP = 47). However, similar to the total scores, when healthy controls were included, moderate to strong significant correlations were found between IPASE and CAARMS subscales.

Our finding that the strength of the correlation depends on whether healthy controls are included suggests that the relationship between IPASE and CAARMS positive symptoms may be more limited within clinical populations, and potentially driven by group-level differences rather than a continuous association. Consistent with previous research into basic symptoms (Müller et al., 2022; Schultze-Lutter and Theodoridou, 2017), this suggests the basic self-disturbance and positive symptoms represent distinct but overlapping domains.

When only the longitudinal subsample of 29 UHR individuals was analysed, there was a significant and strong positive correlation at month-12 follow-up but only a very weak non-significant correlation at baseline. There was also only a weak non-significant correlation between the 12-month change in CAARMS total positive symptom scores and 12-month change in total IPASE scores. These inconsistent findings may have occurred as a result of the reduction in sample size and in the range of CAARMS scores (the longitudinal subsample only included UHR individuals) leading to instability in the correlation. In light of the weak correlation identified at baseline (when healthy controls were excluded), the moderate-to-strong correlation at month-12 is likely to have been a spurious finding. However, this finding could also be explained by individuals developing greater insight into their symptoms after 12 months of treatment, which may enable them to more accurately self-report on anomalous self-experiences. The higher baseline correlation observed in the FEP group compared to the UHR group supports this point. However, the difference in correlations between these subgroups could also be attributed to wide confidence intervals or differences in the variance of CAARMS total positive symptom scores. A recent study found that self-reported IPASE scores were lower in a UHR cohort as compared to a group with first-episode psychosis (Srivastava et al., 2023). However, when the researchers applied a natural language processing technique to open-ended interviews, they found that the spoken language of UHR individuals had greater semantic similarity to IPASE items than that of individuals who have been diagnosed with a psychotic disorder. This suggests that the IPASE self-report scale may have varying sensitivity for illness phases or may be impacted by an individual’s exposure to treatment.

The current study assessed the stability of the IPASE over time to examine the test-retest repeatability of the instrument and to provide insight into whether anomalous self-experiences represent trait- or state-like phenomena. The agreement between baseline and month-12 total IPASE scores was moderate for the IPASE total score and across four of the five IPASE subscales, but poor for the Cognition subscale. These findings are partially consistent with a previous study examining the stability of the EASE total and subscale scores at 7-year follow-up (Koren et al., 2020). The researchers found that EASE total scores and three of the five subscales were moderately stable over time. However, the EASE subscales for demarcation/transitivism and existential reorientation — which have both been found to have the highest correlation with the IPASE demarcation/transitivism subscale (Nelson et al., 2019) — exhibited low stability (Pearson’s *ρ* <0.07). Conversely, the transitivism/demarcation subscale was found to be the most stable subscale in the present study (ICC = 0.64, *ρ* =0.65). This is also in contrast to our initial hypothesis in which we hypothesised that the IPASE transitivism/demarcation subscale would have the lowest stability. Aside from the difference in follow-up duration, one explanation for this is that the EASE and IPASE subscales, whilst both designed to measure the same construct, are based on different items and are not directly interchangeable (Nelson et al., 2019; Cicero et al., 2017).

In both the present and previous studies, inconsistent stability amongst ASE subscales may be explained by the dimensional attributes of self-disturbance phenomena. Several studies have supported the hypothesis that self-disturbance encompasses trait-like as well as state-like aspects in schizophrenia, such that particular dimensions of self-disturbance may be less stable than others (Madeira et al., 2017; Pionke-Ubych et al., 2021; Værnes et al., 2022). However, inconsistent stability amongst subscales could also be explained by poor test-retest reliability for certain components of the IPASE and EASE measurement tools, for example, due to the wording of specific items or variations in the number of items within each subscale.

The current study also assessed whether IPASE scores were predictive of remission of UHR status or change in CAARMS positive symptom scores. Given the small sample size, only a linear and logistic regression model were tested. In contrast to our original hypothesis, and previous work which found EASE to significantly predict positive symptom severity at 12-month follow-up (Værnes et al., 2021), the current study did not find baseline IPASE scores to predict remission or change in CAARMS total positive symptom scores at 12 months. One explanation for this discrepancy in findings could be differences in the study populations. The current study used a small sample of 29 of UHR individuals diagnosed according to positive symptoms or trait-risk. Conversely, Værnes et al. (Værnes et al., 2021) included a slightly larger sample of 38 individuals in their analyses, and included both individuals meeting UHR criteria and those meeting basic symptoms high-risk criteria (COGDIS) (Schultze-Lutter and Theodoridou, 2017). Another potential explanation is the difference in the IPASE and EASE assessment tools, with the EASE being a comprehensive, semi-structured clinical interview conducted by a trained professional, while the IPASE is a brief self-report measure.

The present study findings should be interpreted in the context of several limitations. Firstly, the correlational analyses between the CAARMS and IPASE conducted at baseline included two distinct clinical groups (UHR and FEP), and a secondary analysis further included healthy controls. Caution is warranted in interpreting these findings, as group-level differences may have influenced the observed correlations. Additionally, the CAARMS is primarily designed to assess attenuated psychotic symptoms in UHR populations, and correlations across a mixed sample do not necessarily reflect within-group associations or shared underlying mechanisms. Larger, within-population analyses are needed to clarify the nature and strength of these relationships. Secondly, the longitudinal sample analysed here was small, leading to wide confidence intervals when estimating the agreement and correlation between IPASE scores at two time points. However, sample size estimates for test–retest reliability are typically designed to ensure sufficient power to reject the null hypothesis of zero reliability (Bujang and Baharum, 2017). Given significant test-retest stability was found — even within a small sample — the present study provides preliminary support for the reliability of the IPASE scale. Nonetheless, given the wide confidence intervals and uncertainty around the precise strength of this association, further research in larger cohorts is needed to confirm and better characterise this finding. This small sample did, however, mean that the prediction modelling approach was likely underpowered to reject the null hypothesis that IPASE scores at baseline do not predict a change in positive attenuated symptoms. Additionally, the present study assessed stability of IPASE measurements over a 12-month period, which is relatively short with respect to measuring trait stability. A longer-term study currently underway will allow us to examine whether the present finding of moderate stability extends over a longer time frame (Krcmar et al., 2024). Furthermore, while the short self-report nature of the IPASE facilitates ease of administration, scores and findings should be interpreted with caution given the complexity of self-disturbances and the associated potential for misinterpretation of items. Finally, the longitudinal sample here only included individuals who met UHR for psychosis criteria at baseline, restricting the heterogeneity in clinical scores. Further work on a larger and more clinically heterogeneous sample of individuals is needed to confirm the present findings.

## 5. Conclusions

The present findings suggest that the IPASE is a moderately stable tool that demonstrates potential utility as a brief and accessible assessment tool for anomalous self-experiences. The IPASE measures anomalous self-experiences that appear to be generally correlated with the presence of positive attenuated psychotic symptoms but only weakly correlated with symptom severity. A more comprehensive assessment tool such as the EASE may be necessary to predict the trajectory of positive symptoms in high risk cohorts.

## Supplementary Material

1

## Figures and Tables

**Table 1:** Demographic and baseline clinical characteristics of individuals included in the baseline and longitudinal analyses. Values are presented as mean (SD) or percentage.

	Baseline Sample					Longitudinal Subsample
	HC (*n* = 72)	UHR (*n* = 66)	FEP (*n* = 47)	F-value(*df* = 2)/ *x*^2^(*df* = 2)	Pairwise	UHR (*n* = 29)
Age, Years	21.3 (2.7)	19.2 (3.0)	20.2 (2.7)	10.1*	HC *>* UHR	18.8 (2.7)
Gender, %				3.2		
Male	33.4	43.9	48.9			41.4
Female	66.7	56.1	51.1			58.6
Education						
Total Years	13.8 (2.1)	11.4 (2.3)	11.2 (2.0)	24.1*	HC > UHR, FEP	11.5 (2.4)
Educational Attainment, Percentage						
<HS				24.3*	HC > UHR, FEP	
Graduated HS	12.5	42.4	31.9			41.4
Unknown	76.4	36.4	27.7			34.5
	11.1	21.2	40.4			24.1
Race, Percentage						
White	54.2	74.2	76.6			75.9
Asian	38.9	19.7	12.8		HC	13.8
Black, Hispanic, Other	5.6	3.0	8.5			3.4
Missing	1.4	1.5	2.1			6.9
CAARMS Positive Symptoms Total	2.7 (4.6)	51.8 (17.3)	75.3 (28.2)	265.1*	FEP > UHR > HC	52.1 (16.7)
IPASE Total	75.8 (21.4)	135.3 (39.8)	156.8 (50.1)	79.4*	FEP > UHR > HC	136.8 (41.9)
SOFAS	83.4 (7.5)	62.6 (10.3)	59.5 (14.8)	93.8*	HC > UHR, FEP	63.7 (11.2)
GFS	8.2 (0.7)	6.5 (1.1)	6.3 (1.3)	69.7*	HC > UHR, FEP	6.6 (1.2)
PANSS Subscales						
Positive	7.3 (0.8)	13.2 (2.9)	14.7 (6.1)	73.6*	FEP > UHR > HC	12.3 (2.4)
Negative	7.5 (0.9)	12.4 (5.3)	13.8 (6.1)	35.4*	FEP, UHR > HC	12.4 (5.0)
Antipsychotic Prescription, %	0.0	10.6	85.1	120.5*	FEP > UHR > HC	0.0

* *p* < 0.05.

The F-statistic was used to compare differences in the group means and the chi-squared statistic was used to compare differences in proportions, excluding missing values. *P*-values for pairwise comparisons were adjusted using the Holm method. The longitudinal subsample included individuals with month-12 follow-up data available.

CAARMS, Comprehensive Assessment of at Risk Mental States; HC, healthy control; UHR, ultra-high risk for psychosis; FEP, first episode psychosis; IPASE, Inventory of Psychotic-Like Anomalous Self-Experiences; PANSS, Positive and Negative Syndrome Scale; SCID, Structured Clinical Interview for DSM Disorders; MMD, Major depressive disorder; GAD, Generalised anxiety disorder.

**Table 2:** Test-retest intraclass correlation coefficient and Spearman's rho of the IPASE total and subscale scores between baseline and month-12. Confidence intervals (95 %) provided in brackets.

	Total	Cognition	Self-Awareness and Presence	Consciousness	Somatization	Demarcation/ Transitivism
ICC	0.59 [0.25,0.80]	0.30 [−0.03,0.59]	0.58 [0.28, 0.78]	0.59 [0.30, 0.78]	0.54 [0.22, 0.76]	0.64 [0.27, 0.83]
Spearman’s rho	0.58 [0.24, 0.85]	0.27 [−0.11, 0.61]	0.56 [0.20, 0.81]	0.57 [0.30, 0.77]	0.53 [0.20, 0.77]	0.65 [0.34, 0.88]

**Table 3:** Effect of baseline CAARMS positive scores and IPASE total scores on remission and 12-month change in CAARMS positive scores.

Outcome: Remission					
Variable	Estimate	95 % CI	Wald’s *χ*^2^(*df* = 1)	*P*(> *χ*^2^)	Partial *R*^2^
Intercept	1.72	[−3.24, 7.05]	0.47	0.49	–
Baseline CAARMS Score	−0.10	[−0.21, −0.03]	5.11	0.02*	0.30
Baseline IPASE Score	0.02	[−0.00, 0.05]	2.48	0.12	0.05
Outcome: Change in CAARMS Score					
Variable	Estimate	95 % CI	*|t|*(*df* = 1)	*P*(> |*t*|)	Partial *R*^2^

Intercept	12.54	[−35.44, 60.51]	0.51	0.61	–
Baseline CAARMS Score	−0.21	[−0.80, 0.38]	0.71	0.49	0.06
Baseline IPASE Score	−0.16	[−0.40, 0.07]	1.34	0.19	0.02

Note. CI confidence interval. The binary variable condition is scored as persistent APS 0 and remission 1.

## Appendix A. Supplementary data

Supplementary data to this article can be found online at https://doi.org/10.1016/j.schres.2025.05.003.
