# Supplementary material for: The inventory of psychotic-like anomalous self-experiences (IPASE): Stability and relationships with attenuated psychotic symptoms and remission in individuals at-risk for psychosis
Source: Schizophr Res. Author manuscript; Available in PMC 2026 Jun 21. (PMC13283688; doi:10.1016/j.schres.2025.05.003)
Supplement: 1 [file NIHMS2175140-supplement-1.docx]

**Supplementary Material 1**

Table S1. IPASE Subscale Items

| **Subdomain** | **Items** |
| --- | --- |
| Cognition | 1, 12, 20, 28, 37, 49, 56 |
| Self-Awareness and Presence | 2, 4, 7, 10, 15, 18, 21, 23, 26, 29, 32, 35, 38, 40, 42, 44, 46, 48, 50, 53, 55, 57 |
| Consciousness | 6, 14, 22, 31, 41, 52 |
| Somatization | 3, 5, 8, 11, 13, 16, 19, 24, 27, 30, 33, 36, 39, 43, 47, 51, 54 |
| Demarcation/Transitivism | 9, 17, 25, 34, 45 |

Table S2. Spearman’s correlation between CAARMS positive symptom subscale scores and IPASE subscale scores for baseline sample, including healthy controls. Bootstrapped confidence intervals (95%) provided in brackets.

|  | **IPASE Subscale** | | | | |
| --- | --- | --- | --- | --- | --- |
| **CAARMS Subscale** | **Cognition** | **Self-Awareness and Presence** | **Consciousness** | **Somatization** | **Demarcation/ Transitivism** |
| **Unusual Thought Content** | 0.54 [0.46, 0.66] | 0.61 [0.53, 0.71] | 0.61 [0.53, 0.71] | 0.60 [0.49, 0.71] | 0.55 [0.48, 0.68] |
| **Non-Bizarre Ideas** | 0.52 [0.40, 0.64] | 0.58 [0.48, 0.67] | 0.59 [0.49, 0.68] | 0.54 [0.42, 0.65] | 0.57 [0.46, 0.66] |
| **Perceptual Abnormalities** | 0.57 [0.45, 0.66] | 0.63 [0.43, 0.66] | 0.62 [0.52, 0.72] | 0.61 [0.48, 0.69] | 0.58 [0.40, 0.62] |
| **Disorganised Speech** | 0.56 [0.41, 0.65] | 0.56 [0.50, 0.70] | 0.62 [0.50, 0.70] | 0.59 [0.49, 0.69] | 0.52 [0.43, 0.66] |


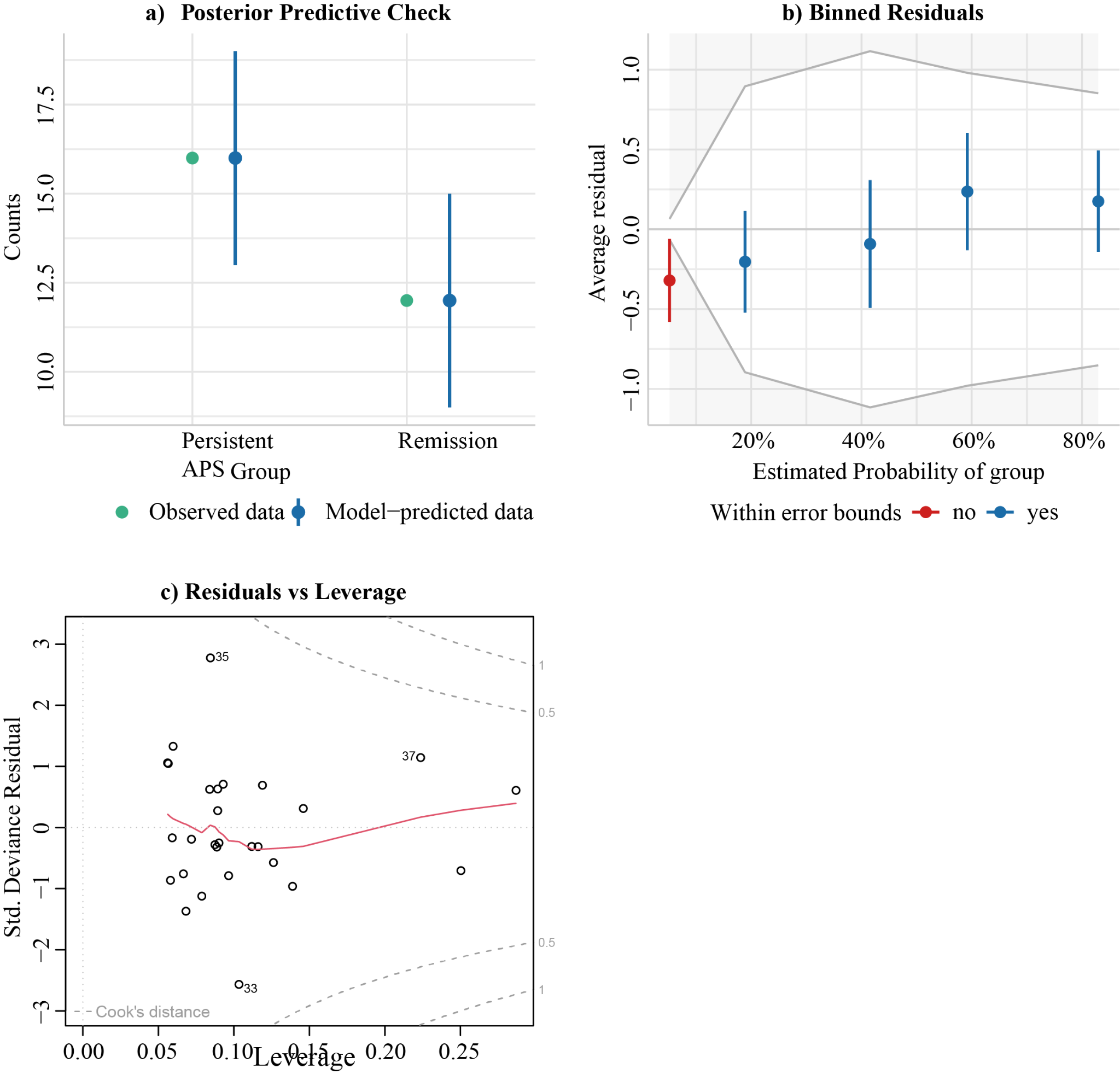


Figure S1. Diagnostic plots for logistic regression model. a) Posterior predictive check (simulated data under fitted model plotted against observed data) shows an acceptable model fit with observed values falling inside the range of predicted values. b) Less than 95% of the residuals fall within the error bounds indicating only an average model fit to the data. c) Residuals vs leverage plot does not indicate the presence of any influential outliers.


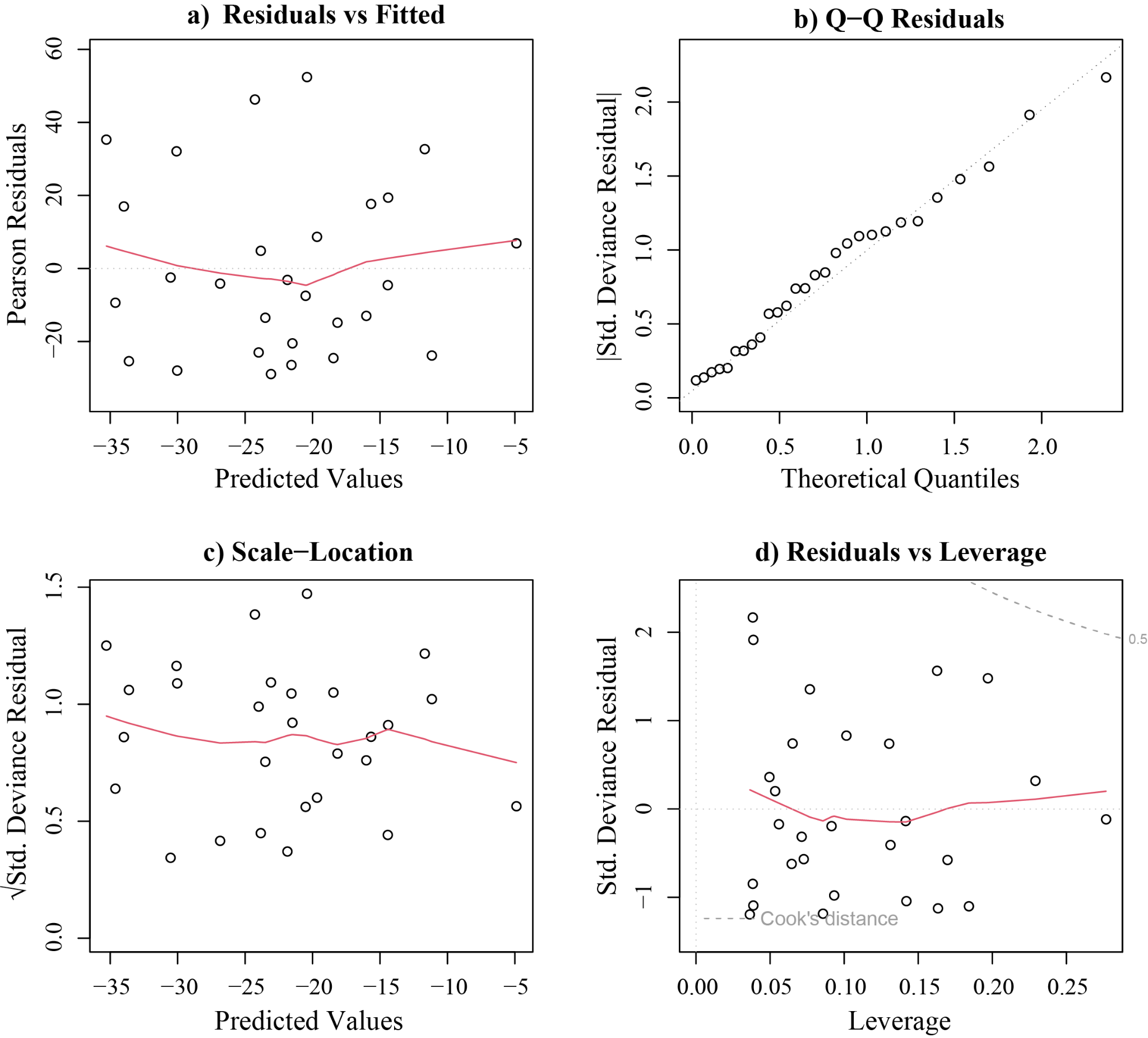


Figure S2. Diagnostic plots for multiple linear regression model after removal of one influential point (n=28). a) No obvious trends in the residuals plotted against fitted values supports the assumption of linearity. No obvious heteroscedasticity is present. b) Q-Q plot showing points closely following the dashed line, indicating no clear violations of normality. c) Standardised residuals plotted against fitted values do not indicate any clear trends, supporting the assumption of homoscedasticity. d) Residuals vs leverage plot does not indicate the presence of any influential outliers (Cook’s distance < 0.5).

Table S3. Effect of baseline CAARMS positive scores and IPASE total scores on 12-month change in CAARMS positive scores after removal of one influential point (n=28).

| **Outcome: Change in CAARMS Score** | | | | | |
| --- | --- | --- | --- | --- | --- |
| **Variable** | **Estimate** | **95 % CI** | ***\|t\|*(*df*=1)** | $\boldsymbol{P(>}\boldsymbol{\vert}\boldsymbol{t}\boldsymbol{\vert}\boldsymbol{)}$ | **Partial** $\boldsymbol{R}^{\boldsymbol{2}}$ |
| Intercept | 10.77 | [−34.24, 55.77] | 0.51 | 0.49 | — |
| Baseline CAARMS Score | -0.45 | [−1.04, 0.14] | 1.49 | 0.15 | 0.08 |
| Baseline IPASE Score | −0.07 | [−0.431, −0.16] | 0.60 | 0.55 | 0.01 |
